# Integrated genomic analyses identify ERRFI1 and TACC3 as glioblastoma-targeted genes

**DOI:** 10.18632/oncotarget.137

**Published:** 2010-08-08

**Authors:** Christopher G. Duncan, Patrick J. Killela, Cathy A. Payne, Benjamin Lampson, William C. Chen, Jeff Liu, David Solomon, Todd Waldman, Aaron J. Towers, Simon G. Gregory, Kerrie L. McDonald, Roger E. McLendon, Darell D. Bigner, Hai Yan

**Affiliations:** ^1^The Preston Robert Tisch Brain Tumor Center and The Pediatric Brain Tumor Foundation and The Department of Pathology, Duke University Medical Center, Durham, NC 27710, USA; ^2^Cancer Genetics Laboratory, Hormones and Cancer Group, Kolling Institute of Medical Research, Royal North Shore Hospital, University of Sydney, St Leonards, NSW, Australia; ^3^Department of Pharmacology & Cancer Biology, Duke University Medical Center, Durham, NC 27710, USA; ^4^Department of Oncology, Lombardi Comprehensive Cancer Center, Georgetown University School of Medicine, Washington, District of Columbia 20057, USA; ^5^Duke Center for Human Genetics, Duke University Medical Center, Durham, NC 27710, USA; ^6^Adult Cancer Program, Prince of Wales Clinical School, Lowy Cancer Research Centre, University of New South Wales, Randwick, NSW, Australia

**Keywords:** glioblastoma, genomics, copy number, 1p36, 4p16, ERRFI1, TACC3

## Abstract

The glioblastoma genome displays remarkable chromosomal aberrations, which harbor critical glioblastoma-specific genes contributing to several oncogenetic pathways. To identify glioblastoma-targeted genes, we completed a multifaceted genome-wide analysis to characterize the most significant aberrations of DNA content occurring in glioblastomas. We performed copy number analysis of 111 glioblastomas by Digital Karyotyping and Illumina BeadChip assays and validated our findings using data from the TCGA (The Cancer Genome Atlas) glioblastoma project. From this study, we identified recurrent focal copy number alterations in 1p36.23 and 4p16.3. Expression analyses of genes located in the two regions revealed genes which are dysregulated in glioblastomas. Specifically, we identify EGFR negative regulator, *ERRFI1*, within the minimal region of deletion in 1p36.23. In glioblastoma cells with a focal deletion of the *ERRFI1* locus, restoration of ERRFI1 expression slowed cell migration. Furthermore, we demonstrate that TACC3, an Aurora-A kinase substrate, on 4p16.3, displays gain of copy number, is overexpressed in a glioma-grade-specific pattern, and correlates with Aurora kinase overexpression in glioblastomas. Our multifaceted genomic evaluation of glioblastoma establishes *ERRFI1* as a potential candidate tumor suppressor gene and *TACC3* as a potential oncogene, and provides insight on targets for oncogenic pathway-based therapy.

## INTRODUCTION

Cancer cells undergo continuous acquisition of heritable genetic variation, manifested as mutations of single base pairs, large or small deletions or insertions, chromosomal translocations, and gain or loss of entire chromosomal regions [1,2]. The accumulation of these somatic genomic changes results in a highly heterogeneous and complex cancer genome. Glioblastoma is the most aggressive and frequently occurring type of brain tumor [3,4]. Recent comprehensive analyses have opened the door to understanding the genetic and molecular alterations that characterize the glioblastoma genome. These studies identified sequence mutations and alterations of DNA copy number, gene expression, and methylation status that may contribute to a distinct network of oncogenic signaling pathways, including receptor tyrosine kinase (RTK), growth factor and phosphatidylinositol-3-OH kinase (PI3K), p53 and retinoblastoma (RB1) pathways [5,6].

Identification of a coding sequence alteration, amplification, or homozygous deletion can help define unequivocal driver genetic alterations in cancers [6]. Several genes in the central glioblastoma pathways were identified through dramatic copy number alterations, such as amplification of the oncogenes, *EGFR, c-MYC, CDK4, PDGFRA, MDM2*, and *MDM4*, and deletion of the tumor suppressor genes, *CDKN2A, CDKN2B*, and *PTEN* [5-9]. Recent studies have validated the significance of well-known copy number alterations and have proposed additional candidates which may contribute to the development of glioblastomas [5,6,10-12]. Conversely, several core glioblastoma genes have been found to be exclusively altered by sequence mutation, such as *PI(3)K*, *RAS*, *ERBB2*, and *IDH1*/*IDH2* [5,13]. However, a large proportion of recognized glioblastoma driver genes, including *EGFR, TP53, CDKN2A, PTEN, NF1* and *RB1*, are targeted by both sequence and copy number alterations. In addition, gene expression signatures, which are an essential component of global genomic studies, have been used to exploit disease-specific signaling pathways and as clinical prognostic factors in many cancers [14-17]. However, despite major advancements, the current understanding of glioblastoma genetics is still inadequate, and additional molecular targets are urgently needed to be used in the development of clinically proven therapies.

In this study, we used Digital Karyotyping (DK) and Illumina BeadChip assays (Illumina, Inc., San Diego, CA) to identify genomic loci that are recurrently targeted by focal copy number alterations in 111 glioblastomas. Using these data and data from TCGA, we identified frequent gene copy number changes in1p36.23 and 4p16.3. We further demonstrated that *ERRFI1* and *TACC3* in 1p36.23 and 4p16.3, respectively, are potential glioblastoma-targeted genes.

## RESULTS

### Detection of focal copy number alterations by DK and Illumina beadchips

DK is a highly quantitative copy number analysis platform that has previously been used to identify copy number events in human cancers, including glioblastomas [18-20]. Analysis of 27 glioblastoma samples revealed 52 high-copy amplification events ranging from 98kb to 6.8Mb with 12 to 205 copies per nucleus. The targeted genes within those regions include gain of *EGFR*, *CDK4*, *PDGFRA*, *MDM2*, and *MDM4* (Supplemental Table S1). In addition, we identified 120 regions of homozygous deletion, ranging from 100kb to 5.1Mb. The most common loss is on chromosome 9p21, where tumor suppressor genes *CDKN2A* and *CDKN2B* are located (Supplemental Table S2).

Illumina BeadChips in conjunction with the Infinium assay have also been effectively used to examine copy number variations in human cancers at high-throughput levels [5,6,20]. First, using the highly quantitative DK data, we optimized the criteria for defining focal high-copy amplifications and homozygous deletions for Illumina high-density SNP arrays. Two glioblastoma samples, xenograft H456 and primary tumor TB2607, were analyzed by both DK and Illumina high-density SNP arrays. Using DK as a standard, we identified the values and filtering criteria (stated in Materials and Methods) that faithfully reveal amplifications and deletions from the data produced by the Illumina BeadChips. We generated Illumina high-density SNP array profiles from 84 glioblastomas samples. As controls, we also analyzed genomic DNA from normal adult and fetal brain and 3 matching blood specimens from glioblastoma patients. In total, we identified 474 focal gain events (Supplemental Table S3) and 1540 focal loss events (Supplemental Table S4).

**Figure 1:**
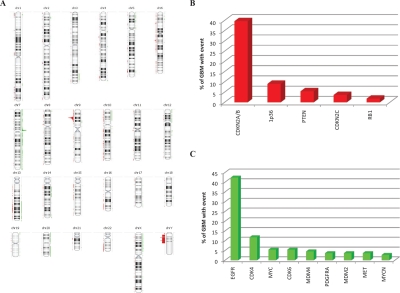
Focal high copy number gains and homozygous deletions in glioblastomas. Copy number analysis using DK and Illumina BeadChips revealed gains (indicated by green) and losses (indicated by red) spanning the genome of glioblastomas. (A) Glioblastoma copy number karyotype generated utilizing Nexus Copy Number Professional Software (BioDiscovery Inc.). The most common regions for (B) focal deletion and (C) focal gain of copy number in 111 glioblastomas. Loci are identified with reported tumor suppressor genes and oncogenes if available.

Of the genomic copy number profiles from a total of 111 glioblastoma samples assessed by DK or Illumina BeadChips, we identified a high degree of heterogeneity and copy number instability across the glioblastoma genomes (Figure 1A). Despite heterogeneity, we detected several regions which are recurrently gained or lost in glioblastomas (Figure 1B, C). The two most prevalent focal amplifications were the *EGFR* and *CDK4* loci, occurring in 42% and 12% of all cases, respectively. The two most prevalent focal deletions were the *CDKN2A/B* and 1p36 loci, occurring in 40% and 9% of all samples, respectively. Additionally, we detected multiple intragenic homozygous deletions within large genes, including *LRP1B*, *WWOX*, and *A2BP1*.

### ERRFI1 on 1p36 is a candidate tumor suppressor gene, whose products regulate glioblastoma cell migration

**Figure 2:**
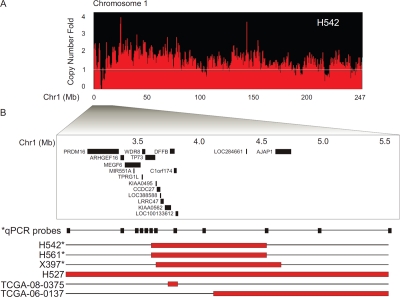
High-resolution mapping of homozygous deletions detects *DFFB, C1orf174*, and *LOC100133612* within secondary MDr on 1p36.32. (A) Chromosome 1 DK analysis of glioblastoma cell line H542 indicates a single homozygous deletion occurring at the 1p36.32 cytoband. (B) High-resolution mapping depicts overlapping homozygous deletions from 3.0Mb to 5.5Mb coordinates on chromosome 1 (indicated by red bars). Copy number data includes tumor samples from our independent analysis plus the TCGA glioblastoma copy number data set. RefSeq gene positions are indicated. Genomic Q-PCR validation analysis (*) of H542, H561, and glioblastoma xenograft X397 confirms homozygous deletion. Q-PCR primer location depicted by black boxes (Supplemental Table S5).

**Figure 3:**
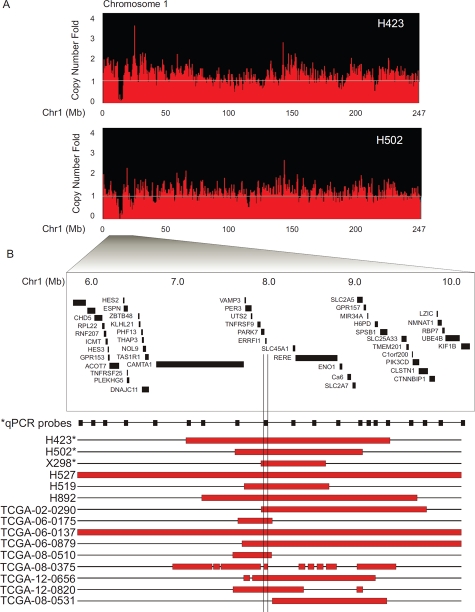
High-resolution mapping of homozygous deletions reveals *ERRFI1* within the most frequent MDr on 1p36.23. (A) DK analysis of chromosome 1 in glioblastoma cell lines H423 and H502 reveals a single homozygous deletion at 1p36.23. (B) High resolution mapping depicts overlapping homozygous deletions from 6.0Mb to 10.0Mb coordinates on chromosome 1 (indicated by red bars). Copy number data include tumor samples from our independent analysis and the TCGA glioblastoma copy number data set. RefSeq gene positions are indicated. Validation by genomic Q-PCR copy number analysis (*) of H423, H502, and glioblastoma xenograft X298 confirms homozygous deletion. Q-PCR primer location depicted by black boxes (Supplemental Table S6).

In addition to the well-characterized glioblastoma genes, the most striking genomic changes identified in our analysis were recurrent focal copy number changes on 1p36 and 4p16. Focal deletions of 1p36 have been previously reported to occur in numerous cancer types, including glioblastoma, neuroblastoma, oligodendroglioma, and colorectal, lung, and breast cancer [21-23]. We detected focal homozygous deletions on 1p36 in 9/111 of our glioblastoma samples. With the addition of samples from the TCGA glioblastoma data set, we mapped 1p36 deletions in a comprehensive data set of a total 430 glioblastomas and found they occurred in two distinct minimal deleted regions (MDRs) on 1p36.32 (Figure 2) and on 1p36.23 (Figure 3).

Glioblastoma cell line H542 presented a homozygous deletion at 1p36.32 (Figure 2A). We mapped the 1p36.32 deletion in H542 by quantitative real-time PCR (Q-PCR) and found that this region contained *TP73*, *KIAA0495*, *CCDC27*, *LOC388588*, *LRRC47*, *KIAA0562*, *DFFB*, *C1orf174*, *LOC100133612*, and *LOC284661* (Figure 2B, Supplemental Table 5). Further analysis of additional samples with 1p36.32 deletions revealed an overlapped MDR containing *DFFB*, *C1orf174*, and *LOC100133612* (Figure 2B).

Cytoband 1p36.23 harbors the most frequently deleted region (Figure 3) on 1p36 in glioblastomas. Two glioblastoma cell lines, H423 and H502, each contained a single focal deletion in 1p36.23 (Figure 3A). We further mapped the 1p36.23 deletions in the two cell lines by Q-PCR. The deleted region contains multiple genes, including *CAMTA1*, *VAMP3*, *PER3*, *UTS2*, *TNFRSF9*, *PARK7*, *ERRFI1*, *SLC45A1*, *RERE*, *ENO1*, *CA6*, *SLC2A7* and *SLC2A5* (Figure 3B, Supplemental Table 6). With the addition of TCGA glioblastoma samples, we found that 15/430 glioblastomas contain homozygous deletions in 1p36.23. Further analysis revealed the most commonly deleted region contains a single gene, *ERRFI1* (Figure 3B).

**Figure 4:**
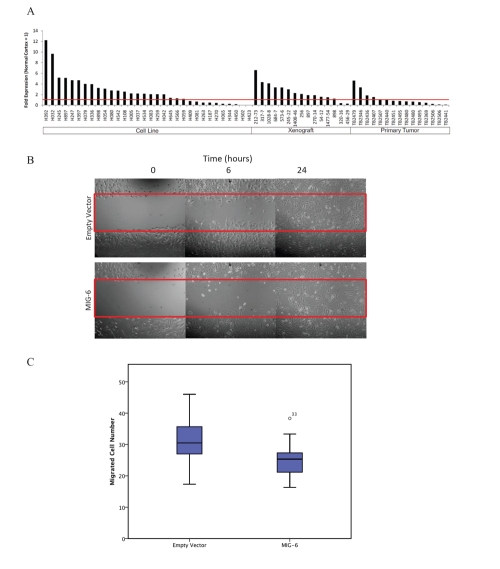
*ERRFI1* is silenced in glioblastomas and reduces cell migration in H423 glioblastoma cells. (A) Quantification of *ERRFI1* mRNA levels in glioblastomas (32 cell lines, 15 xenografts, and 15 primary tumors) by Q-PCR. Results presented as fold expression relative to normal cortex control (indicated by red line). Restoration of ERRFI1 expression in H423 cells results in decreased cell migration as measured by (B) wound healing assay and (C) trans-well migration assay (T-test, p < 0.001).

*ERRFI1* is a candidate tumor suppressor which functions in normal cells as a negative regulator of EGFR and the ErbB family [24-26]. We measured *ERRFI1* mRNA levels in a panel of 62 glioblastoma samples and detected downregulation of ERRFI1 expression in 34% of the samples tested (Figure 4A).

We further investigated the possible pathogenic function of ERRFI1 in glioblastoma cells*.* Glioblastoma cell line H423 harbors a homozygous deletion of *ERRFI1* and did not express endogenous ERRFI1 (Figure 4A). We transfected H423 cells with pCMV-6-entry-ERRFI1 and performed wound-healing experiments on the transfected cells. Compared to cells transfected with a control vector, H423 cells with ERRFI1 expression had impaired wound healing (Figure 4B). Furthermore, we conducted trans-well assays and demonstrated that H423 cells with ERRFI1 expression had a lower rate of trans-well migration than control cells (Figure 4C).

### TACC3 on 4p16.3 is overexpressed and correlates with the expression of Aurora kinases in glioblastomas

**Figure 5:**
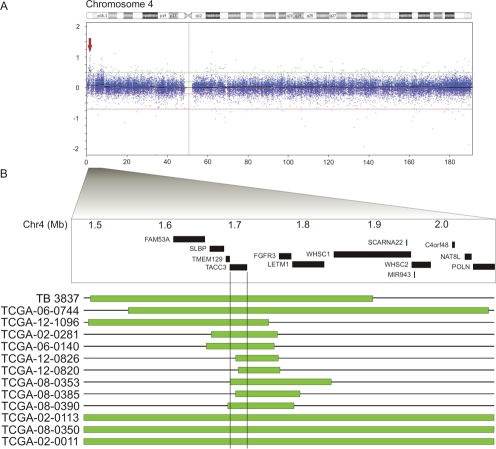
High resolution mapping identifies *TACC3* as the glioblastoma-targeted gene on 4p16.3. (A) Illumina Bead-Chip analysis of chromosome 4 in glioblastoma primary tumor TB3837 reveals a single focal gain at 4p16.3. (B) High resolution mapping depicts overlapping gain of copy number from 1.5Mb to 2.1Mb coordinates on chromosome 4 (indicated by green bars). Copy number data includes tumor samples from our independent analysis and the TCGA glioblastoma copy number data set. RefSeq gene positions are indicated.

**Figure 6:**
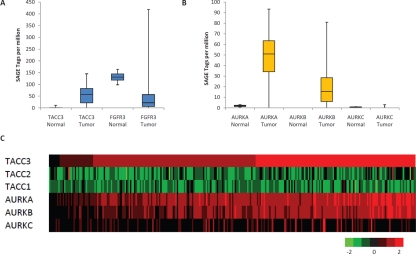
*TACC3* is the predominant gene upregulated in 4p16.3 and correlates with Aurora kinase expression. SAGE of (A) TACC3 and FGFR3 and (B) Aurora kinases, AURKA, AURKB, and AURKC in normal brain (n = 2) and glioblastoma (n = 16) samples (data adapted from [5]). (C) Gene expression of TACC and Aurora kinase family members using the TCGA glioblastoma expression data set (n = 266) indicates correlation between TACC3 and AURKA (correlation coefficient = 0.76) and between TACC3 and AURKB (correlation coefficient = 0.70). Red indicates high gene expression level and green indicates low gene expression level.

Focal gains on 4p16.3 were also frequently identified in a subset of glioblastomas (Figure 5A). We mapped the 4p16 duplications in a data set of 430 glioblastomas. The gains clustered in a minimal gained region centered on 4p16.3 from 1.5 to 2.0Mb (Figure 5B), containing *SLBP*, *TMEM129*, *TACC3*, *FGFR3* and *LETM1*, of which *TACC3* most commonly displayed gain of copy number. A Q-PCR analysis of an independent panel of glioblastoma samples detected genomic duplications of *TACC3* in 5 out of 101 samples. In addition, compared with its adjacent genes, *SLBP*, *TMEM129* and *FGFR3* at 4p16.3, we found that *TACC3* displayed a predominant overexpression pattern in glioblastomas (Figure 6A, S1 D).

**Fig 7.**
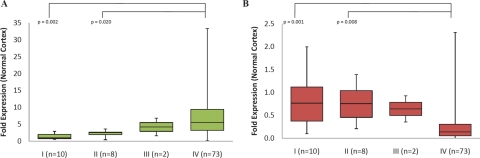
Grade-specific TACC3 upregulation and TACC2 downregulation in gliomas. (A) *TACC3* and (B) *TACC2* mRNA levels in a panel of 93 glioma samples were assessed by Q-PCR. Results presented as fold expression relative to normal cortex control. I (WHO classification glioma Grade I), II (WHO classification glioma Grade I), III (WHO classification glioma Grade III), IV (WHO classification glioma Grade IV).

Dysregulation of the human Transforming Acidic Coiled Coil (TACC) genes is thought to be important in the development of several cancers. The three human TACC proteins, TACC1, TACC2, and TACC3, are core components of the centrosome and have non-overlapping functions in the normal cell and the cancer cell [27,28]. TACC1 overexpression promotes cellular transformation *in vitro* [29] and *in vivo* [30]. TACC2 (AZU-1) is a putative tumor suppressor in breast cancer [31]. TACC3 expression is upregulated and associated with shorter median survival in patients with non-small-cell lung cancer [32]. However, TACC3 expression is reduced in ovarian and thyroid cancer [33,34]. We therefore assessed expression of the TACC genes in a panel of gliomas (WHO Grades I-IV) by Q-PCR (Figure 7). We found significant overexpression of TACC3 in Grade IV gliomas. Conversely, TACC2 transcript levels were significantly downregulated in Grade IV gliomas.

TACC3 can be phosphorylated by Aurora-A kinase and plays an important role in mitosis [35-37]. Therefore, to determine the correlation of TACC3 and Aurora kinase expression, we used Serial Analysis of Gene Expression (SAGE) [5] and TCGA expression data sets to evaluate TACC3 and Aurora kinase gene expression in glioblastomas (Figure 6B, 6C). We found a significant expression correlation between *TACC3* and Aurora A (correlation coefficient = 0.76), as well as between *TACC3* and Aurora B (correlation coefficient = 0.70) (Figure 6C).

## DISCUSSION

While many of the most common copy number gains and deletions have been characterized in glioblastomas, the significance of many other genomic events remains unknown. Utilizing an independent set of glioblastomas and publicly available data, in this study, we focused on the identification and characterization of genes on two frequently altered regions, 1p36 and 4p16. We found that *ERRFI1* is a potential glioblastoma-targeted tumor suppressor gene and *TACC3* is a potential oncogene.

Deletions in 1p36 have been previously reported in glioblastoma genomic studies, indicating that multiple tumor suppressors exist in this locus [19,38-42]. In our analysis, we observed several regions of deletions across 1p36. However, the most striking and frequent deletions occur between the 5 and 10Mb coordinates on 1p36.23, centering on the candidate tumor suppressor *ERRFI1*. Deletion of *ERRFI1* has been reported to activate EGFR and sustain MAPK signaling, resulting in tumor phenotypes in numerous tissues in *ERRFI1* knockout mice [43-45]. *ERRFI1* is also frequently deleted, mutated, or down-regulated in breast and lung cancers, as well as in glioblastomas[38,42,46]. In a recent study, overexpression of *ERRFI1* was shown to decrease proliferation in glioblastoma cells, binding EGFR with STX8, and driving internalized EGFR to late endosomes for degradation, whereas knockdown of ERRFI1 expression resulted in increased tumor invasion [47]. Our biological data are consistent with these previous findings, showing that restoring ERRFI1 expression in an ERRFI1-deficient glioblastoma cell line decreases glioblastoma cell migration. Our genetic and biological data, as well as data from other studies, suggests that ERRFI1 is another key component in the EGFR signaling pathway involved in glioblastoma development.

Genomic alterations of 4p16.3 have been reported in several cancers, indicating the presence of one or more oncogenes [48-51]. Using an integrative genomic strategy, we have analyzed genes altered in the 4p16.3 region in glioblastomas and revealed that *TACC3* is the primary glioblastoma-targeted gene in this region. Furthermore, a *TACC3* somatic mutation (p.E680K) was reported in one of 22 glioblastomas [5]. *TACC3* has a conserved function to promote centrosomal microtubule assembly, a process which is often altered in cancer cells [27,52]. Consequently, genetic silencing of TACC3 results in destabilized microtubules, defects in chromosome alignment, and mitotic defects [52-54].

The Aurora family of serine-threonine kinases is comprised of three members (A, B, and C) that cooperate with many other proteins, including the TACC family, to direct chromosome assembly and segregation during mitosis [27,55-57]. Dysfunction of Aurora kinases can disrupt genomic integrity and lead to aneuploidy, mitotic arrest, and cell death. Aurora A and B are of particular interest since they have been shown to be overexpressed in a broad range of human tumors and are often associated with poor outcome. Thus, regulators of the mitotic spindle apparatus, including Aurora kinases and its substrates, are attractive targets for small-molecule therapeutics [35,58-60]. In our study, we found a strong correlation between Aurora kinase and TACC3 expression, indicating that in the Aurora kinase/TACC pathway, the dysregulated kinase and its substrate, may contribute synergetically to glioblastoma pathogenesis and could serve as targets for molecular-based intervention.

## MATERIALS AND METHODS

### Tumor samples

DNA samples were obtained from brain tumor cell lines, xenografts and primary brain tumors. Brain tumor tissue samples were obtained from the Preston Robert Tisch Brain Tumor Center Biorepository at Duke University Medical Center by an IRB-approved protocol. All samples were obtained in accordance with the Health Insurance Portability and Accountability Act. Frozen sections were made from each tumor sample and examined by light microscopy by a board-certified neuropathologist to ensure that more than 95% of each section consisted of tumor cells. Normal patient DNA samples were obtained from peripheral blood. Normal adult and fetal brain genomic DNA was obtained from BioChain Institute, Inc. (Hayward, CA). Samples were defined as pediatric if taken from a patient between the ages of 0 and 19.

### Digital Karyotyping

We generated 18 glioblastoma DK libraries. Additionally, we included 1 primary and 8 glioblastoma cell line DK libraries from the Cancer Genome Anatomy Project [19] (http://cgap.nci.nih.gov/SAGE/DKViewHome). Protocols for performing DK and software for the extraction and analysis of genomic tags are available at www.digitalkaryotyping.org [61]. Experimental tag sequences were compared to predicted human genome virtual tags and were visualized by using SageGenie DKView (http://cgap.nci.nih.gov/SAGE/DKViewHome). Homozygous deletions were screened by using a sliding window size of 150 virtual tags (~600 kb in size). Putative homozygous deletions were defined as events with a tag density ratio (observed tags/expected tags in window) of < 0.05. Amplifications were identified using a sliding window size of 50 virtual tags (~200 kb in size). Putative amplifications were defined as events with a tag density ratio of > 6.

### High density SNP arrays

Eighty-four glioblastoma samples were analyzed for copy number variation by utilizing HumanHap550-Duo and Human610-Quad BeadChips with the Illumina Infinium Whole Genome Genotyping Assay. Data was pre-processed to generate logR intensity ratios using Illumina BeadStudio. Data was converted into copy number calls and visualized by using Nexus Copy Number software (BioDiscovery, Inc., El Segundo, CA).

### TCGA Data

Genome-wide Level 3 TCGA copy number data was downloaded with the TCGA Data Portal Data Access Matrix (http://cancergenome.nih.gov/dataportal) from Hudson-Alpha Cancer Genome Characterization Center, Memorial Sloan Kettering Cancer Center, and Harvard Medical School-Dana Farber Cancer Institute. Candidate deletions were defined as events with seg mean ≤ −1. Candidate gains were defined as events with seg mean ≥ 0.4. Events less than 30kb and events occurring in two or more tumors with identical start and stop positions were removed, because they were likely artifact or copy number polymorphisms. Candidate gene TCGA expression data from the University of North Carolina and Broad Institute was downloaded with the TCGA Data Portal Data Browser. Gene expression values from the TCGA database represent the ratio of tumor expression to normal expression. The expression value for a given gene for a given patient is the log2 ratio of the tumor expression of the gene in the patient to a synthetic normal sample.

### Bioinformatic Analysis

Nexus Copy Number software (BioDiscovery, Inc) was used to visualize copy number data. The following values were used for screening of genetic events of homozygous loss and amplification: Significance Threshold, 1×10^−8^; Min probes per segment, 2; Max contiguous probe spacing (Kbp), 1000; Gain, 0.5; Loss, −0.7. For identification of areas of duplication, the Nexus default settings were as follows: Significance threshold, 1×10^−6^, Min probes per segment, 5; Max contiguous probe spacing (Kbp), 1000; Gain, 0.2.

### Quantitative Real-Time PCR

The genomic DNA content and mRNA expression levels of genes of interest within tumor and normal cells were quantified by quantitative real-time polymerase chain reaction (Q-PCR). Genomic DNA from normal blood cells served as controls, and genomic DNA content was normalized to that of Line-1. For mRNA expression measurement, cDNA from normal human adult cortex was used as the control and cDNA content was normalized to that of GAPDH. Primers for genomic mapping of 1p36.32 and 1p36.23 are listed in Supplemental Table S5 and Supplemental Table S6.

### Wound Healing Assay

H423 glioblastoma cells were maintained in ZO+ media (Gibco, Carlsbad, CA) with 10% fetal calf serum (FCS). Cells were transfected with pCMV6-entry or pCMV6-ERRFI1. Cells were plated at 100% confluence in 6-well plates. The cell monolayer was wounded with a p1000 pipette tip and wound healing was documented at 0, 6 and 24 hours by photographs.

### Transwell Migration Assay

H423 cells were transfected with pCMV6-entry or pCMV6-ERRFI1, washed, and resuspended in serum-free media, then seeded into transwell membranes at 10,000 cells per well with 5% FCS as a chemoattractant. After 24 hours, cells were removed from the upper chamber then fixed in 100% methanol on the lower surface of the membrane and stained with crystal violet (2% solution in ethanol) and counted (experiment performed in triplicate wells, 12 fields counted per well). Statistical analyses were conducted with PASW (SPSS) 18.0 (IBM, Armonk, North Castle, NY).

## SUPPLEMENTAL FIGURE AND TABLES

Supplemental Figure 1

Supplemental Table 1

Supplemental Table 2

Supplemental Table 3

Supplemental Table 4

## Abbreviations used:

TACC3: transforming, acidic coiled-coil containing protein 3
FGFR3: fibroblast growth factor receptor 3
AURKA: aurora kinase A
AURKB: aurora kinase B
AURKC: aurora kinase C
ERRFI1: ERBB receptor feedback inhibitor 1
EGFR: epidermal growth factor receptor
ERBB: epidermal growth factor receptor family
TCGA: The Cancer Genome Atlas
CNV: copy number variation
SAGE: Serial Analysis of Gene Expression
Q-PCR: quantitative polymerase chain reaction
FCS: fetal calf serum.
