# Supplementary figures and images for: Integrated genomic analyses identify ERRFI1 and TACC3 as glioblastoma-targeted genes
Source: Oncotarget. 2010 Aug 8;1(4):265–77. doi: 10.18632/oncotarget.137 (PMC2992381; doi:10.18632/oncotarget.137)

**A**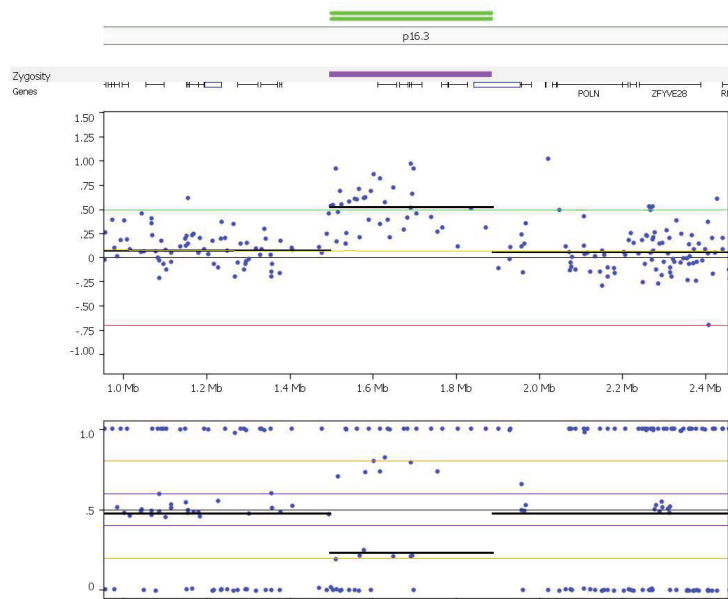

TB3837: Chromosome 4

**B**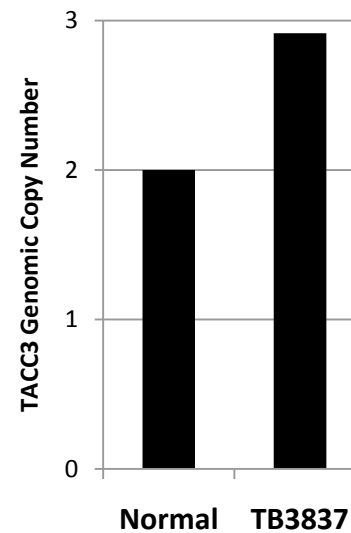**C**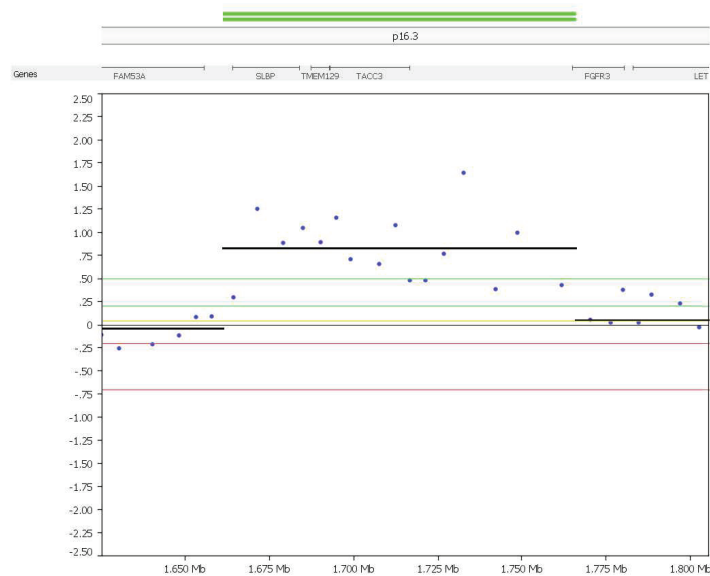

TCGA-02-0281-01A-01D-0276-04: Chromosome 4

**D**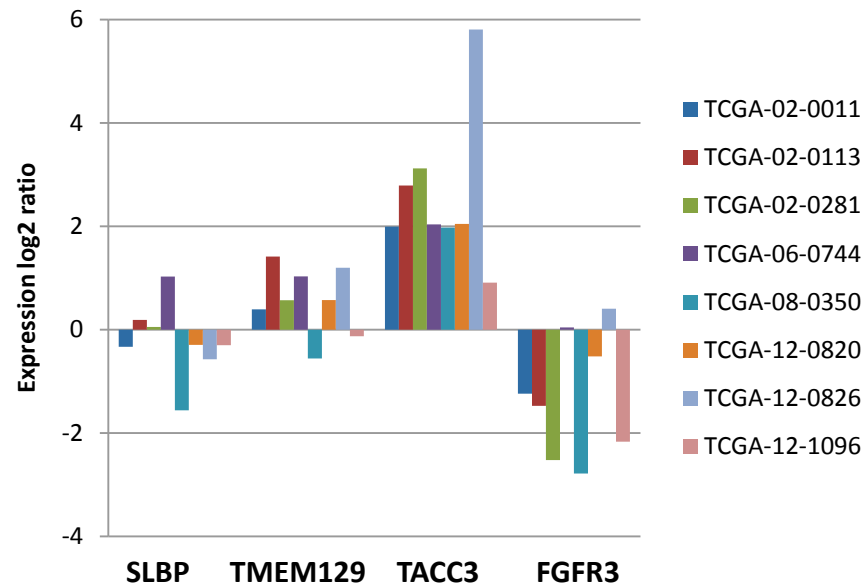

Supplement: Supplemental Figure 1 [file oncotarget-01-265-s001.pdf]
